# Curcumin Suppresses the Lipid Accumulation and Oxidative Stress Induced by Benzo[a]pyrene Toxicity in HepG2 Cells

**DOI:** 10.3390/antiox10081314

**Published:** 2021-08-20

**Authors:** Seung-Cheol Lee, Seung-Cheol Jee, Min Kim, Soee Kim, Min Kyoung Shin, Yunkyung Kim, Jung-Suk Sung

**Affiliations:** Department of Life Science, Dongguk University-Seoul, Goyang 10326, Korea; hen4118@gmail.com (S.-C.L.); markjee@naver.com (S.-C.J.); pipikimmin@naver.com (M.K.); soeesoee@naver.com (S.K.); samantha1994@naver.com (M.K.S.); 252114@naver.com (Y.K.)

**Keywords:** curcumin, lipid accumulation, B[a]P toxicity, ROS, CYP1A1, CYP1B1

## Abstract

Benzo[a]pyrene (B[a]P) is a potentially hepatotoxic group-1 carcinogen taken up by the body through ingestion of daily foods. B[a]P is widely known to cause DNA and protein damages, which are closely related to cell transformation. Accordingly, studies on natural bioactive compounds that attenuate such chemical-induced toxicities have significant impacts on public health. This study aimed to uncover the mechanism of curcumin, the major curcuminoid in turmeric (*Curcuma longa*), in modulating the lipid accumulation and oxidative stress mediated by B[a]P cytotoxicity in HepG2 cells. Curcumin treatment reduced the B[a]P-induced lipid accumulation and reactive oxygen spicies (ROS) upregulation and recovered the cell viability. Cytochrome P450 family 1 subfamily A polypeptide 1 (CYP1A1) and Cytochrome P450 subfamily B polypeptide 1 (CYP1B1) downregulation resulting from decreased aryl hydrocarbon receptor (AhR) translocation into nuclei attenuated the effects of B[a]P-induced lipid accumulation and repressed cell viability, respectively. Moreover, the curcumin-induced reduction in ROS generation decreased the nuclear translocation of Nuclear factor erythroid-2-related factor 2 (Nrf2) and the expression of phase-II detoxifying enzymes. These results indicate that curcumin suppresses B[a]P-induced lipid accumulation and ROS generation which can potentially induce nonalcoholic fatty liver disease (NAFLD) and can shed a light on the detoxifying effect of curcumin.

## 1. Introduction

Curcumin, a natural compound classified in the category of curcuminoids, is mainly found in turmeric (*Curcuma longa*), which is used as an herbal supplement, cosmetic, and food flavor. Recent studies have mainly highlighted its effects in the prevention of obesity and diabetes, suppression of the accumulation of fatty acids in the liver, and attenuation of inflammation by downregulating inflammatory cytokines and its role as an effective antioxidant that upregulates antioxidant enzymes that consequently reduce reactive oxygen species (ROS) levels [1,2,3,4,5,6].

Recent studies have focused on natural bioactive compounds that are derived from functional foods and attenuate chemical-induced toxicities [2,7,8]. Benzo[a]pyrene (B[a]P) is widely known as a chemical carcinogen of polycyclic aromatic hydrocarbon, listed as a group-1 carcinogen by the International Agency for Research on Cancer (IARC) [9,10,11]. The harmful effects of B[a]P on human health include induction of DNA and protein damages, such as 8-oxodG, DNA adducts, and protein adducts, which finally cause carcinogenic transformation of cells [12,13,14,15]. The polluted air from cigarette smoke, engine exhaust, or domestic heating contains B[a]P, and intake of B[a]P through inhalation of such polluted air not only increases the risk of developing lung cancer but also is related to other types of cancer [16,17,18,19,20]. Similarly, epidemiological studies have shown that environmental exposure to B[a]P increases the risk of liver cancer, and B[a]P-rich foods, such as smoked, barbecued, fried, or overcooked meat products, increase the risk of breast cancers [21,22,23,24,25,26]. Since such a natural and chronic exposure of B[a]P is inevitable in contemporary societies, research on the attenuation of the toxicity of B[a]P is important. B[a]P ingested through dietary exposure is metabolized into epoxides, dihydrodiols, phenols, and quinones, which generate ROS in the liver, and it is also known that B[a]P induces hepatic steatosis by increasing the adiposity of the liver [27,28]. Accumulation of lipids in hepatocytes causes hepatic steatosis and, consequently, the susceptibility of the liver to chronic cell death, leading to steatohepatitis, which can potentially progress into fibrosis and cancer [29,30,31]. Hepatic steatosis and steatohepatitis are both classified under the term “nonalcoholic fatty liver disease” (NAFLD) when the causes of the disease are not related to alcohol consumption [29]. B[a]P is known to be a direct ligand of aryl hydrocarbon receptor (AhR), which is a basic helix–loop–helix/Per-ARNT-Sim (bHLH-PAS) transcription factor [32,33]. The activation of AhR turns on the expression of cytochrome P450 family 1 subfamily A polypeptide 1 (*Cyp1a1*) and subfamily B polypeptide 1 (*Cyp1b1*). These genes are known for their activities in the metabolism of xenobiotics and function in the generation of ROS [34,35,36,37,38]. Recent studies have shown that especially CYP1A1 acts as a primary regulator in AhR-mediated lipid production, thereby causing lipid accumulation, and CYP1A1 expression, stimulated by ROS, also contributes to the accumulation of lipid droplets in hepatocytes [39,40]. Taken together, B[a]P binding to AhR induces lipid accumulation, which can cause NAFLD, through the CYP1A1-mediated pathway and induces ROS generation by upregulating CYP1A1 and CYP1B1 [35,39]. In this study, we focused on the suppressive effect of curcumin on B[a]P-induced lipid and ROS accumulation in HepG2 cells and the underlying mechanisms.

## 2. Materials and Methods

### 2.1. Chemicals and Reagents

Fetal bovine serum (FBS), minimum essential medium (MEM), penicillin/streptomycin, trypsin-ethylene, and sodium pyruvate were purchased from Welgene (Daegu, Korea). Curcumin, B[a]P, phenylmethylsulfonyl fluoride, 4,6-diamidino-2-phenylindole dihydrochloride (DAPI), formalin, isopropanol, dichlorofluorescein diacetate, oil red O (ORO), and Triton X-100 were purchased from Sigma-Aldrich Chemical (St. Louis, MO, USA). The mounting medium for immunocytochemical analysis was purchased from Dako (Carpinteria, CA, USA). Antibodies against Nrf2 (sc-365949), AhR (sc-133088), CYP1A1 (sc-20772), CYP1B1 (sc-32882), NQO1 (sc-32793), m-IgGκ BP-HRP (sc-516102) and β-actin (sc-47778), siRNAs against *Cyp1a1* and *Cyp1b1* as well as the control siRNA were purchased from Santa Cruz Biotechnology (Santa Cruz, CA, USA). Anti-Catalase antibody (12980S), Anti-rabbit IgG HRP-linked Antibody (7074S), Anti-mouse IgG (H+L) F(ab’)_2_ Fragment Alexa Flour 488 antibody (4408S) and Anti-mouse IgG (H+L), F(ab’)2 Fragment Alexa Flour 555 antibody (4409S) was purchased from Cell Signaling Technology (Danvers, MA, USA).

### 2.2. Cell Culture

HepG2 cells (American Type Culture Collection, Manassas, VA, USA) were cultured at 37 °C in a 150 mm cell-culture dish with MEM containing 10% FBS, 100 μg/mL penicillin/streptomycin, and 1 mM sodium pyruvate (Welgene, Gyeongsan, Korea) in a humidified incubator with 5% CO_2_. Stock solution for curcumin and B[a]P was made at the concentration of 20 mM diluted in DMSO. Cells were treated with B[a]P and curcumin at the final concentration of 10 µM in cell-culture media for 48 h.

### 2.3. Cell Viability Assay

Cell viability assay was performed to evaluate the cytotoxicities of B[a]P and curcumin (Sigma-Aldrich Chemical, St. Louis, MO, USA) on HepG2 cells. The concentration (10 µM) of B[a]P was selected based on the evidence reported in our previous study which showed cytotoxicity of the B[a]P occurring in a dose-dependent manner on HepG2 cells [8]. HepG2 cells were seeded at a density of 1 × 10^4^ cells/well in 96-well plates containing MEM with B[a]P (10 µM) or curcumin (0, 1, 5, 10, 20, or 40 µM) and cultured for 48 h. The cells were then incubated with EZ-CYTOX reagent (DOGEN, Seoul, Korea) for an additional 2 h. Cell absorbance at 450 nm was measured using a microplate reader (Tecan, Männedorf, Switzerland), and the cell viability with or without the treatment of B[a]P or curcumin was assessed.

### 2.4. Assessment of Intracellular ROS Level

The intracellular ROS levels were evaluated using 2′,7′-dichlorofluorescein diacetate (DCF-DA) (Sigma-Aldrich Chemical, St. Louis, MO, USA). In cells, this chemical is deacetylated to 2′,7′-dichlorodihydrofluorescein (DCF), which fluoresces upon oxidation by intracellular ROS. HepG2 cells (5 × 10^3^ cells/well) were seeded in 96-well plates and treated with B[a]P and curcumin. Cells were then washed with PBS and incubated in a humidified incubator with 10 µM DCF-DA in PBS for 30 min. Intracellular ROS levels were assessed using a fluorescence microplate reader (Tecan, Männedorf, Switzerland) at 485/535 nm.

### 2.5. ORO Staining

For the evaluation of triglyceride vesicles in HepG2 cells via ORO staining, cells were washed twice with PBS and fixed with 10% formalin for 10 min on ice. After the fixation, the cells were washed with PBS and then 60% isopropanol. They were then air-dried at room temperature. The cells were stained with ORO (Sigma-Aldrich Chemical, St. Louis, MO, USA) at room temperature for 30 min and then washed twice with distilled water. To quantify the ORO stain in the cells, 100% isopropanol was used to dissolve the dye in the cells, and the absorbance at 492 nm was measured by using a microplate reader (Tecan, Männedorf, Switzerland).

### 2.6. Measurement of Triglyceride and Free-Glycerol Levels

The total triglyceride levels in HepG2 cells and the free-glycerol levels in cell-culture media were quantified using the Triglyceride Quantification Kit (BioVison Inc., Milpitas, CA, USA) and Free Glycerol Assay KIT (BioVison Inc., Milpitas, CA, USA), respectively, according to the protocols of the manufacturers.

### 2.7. Immunofluorescence Staining

HepG2 cells were seeded in a 6-well cell-culture plate and incubated for a day. They were then fixed with 4% formaldehyde for 15 min and treated with 0.25% Triton X to permeabilize all lipid bilayers, including the nuclear membrane. The cells were then treated with the primary antibody against AhR or Nrf2 (Santa Cruz Biotechnology, Santa Cruz, CA, USA) and incubated overnight at 4 °C. Then, the cells were treated with the Alexa 488-conjugated or Alexa 555-conjugated anti-mouse secondary antibody for 45 min (Cell Signaling Technology, Danvers, MA, USA). DAPI was used to counterstain the nuclei. Confocal fluorescent images were obtained using a confocal microscope (Carl Zeiss, Oberkochen, Germany), and fluorescence was quantified using ImageJ (National Institutes of Health, Bethesda, MD, USA). The ‘relative fluorescence intensity’ was quantified with the calculation (intracellular fluorescence intensity/DAPI intensity) and the ‘relative fluorescence levels in nuclei’ with the calculation (fluorescence intensity in nuclei/DAPI intensity).

### 2.8. siRNA Transfection

Before transfection, HepG2 cells were seeded in a 6-well cell-culture plate and incubated for 24 h until they reached 80% confluence. *Cyp1a1* and *Cyp1b1* were knocked down using CYP1A1 and CYP1B1 siRNAs (Santa Cruz Biotechnology, Santa Cruz, CA, USA), respectively, by following the manufacturer’s protocol. Control siRNA (Santa Cruz Biotechnology, Santa Cruz, CA, USA) was used as the vehicle control, and the cell lysates were subjected to Western blot analysis to evaluate the knockdown efficiency.

### 2.9. Western Blot Analysis

HepG2 cells were lysed using a lysis buffer containing the RIPA buffer (Bio Solution, Korea), phenylmethylsulfonyl fluoride (Sigma-Aldrich Chemical, St. Louis, MO, USA), a protease inhibitor (Sigma-Aldrich Chemical, St. Louis, MO, USA), and a phosphatase-inhibitor cocktail 2/3 (Sigma-Aldrich Chemical, St. Louis, MO, USA). The lysates were then centrifuged at 25,000× *g* for 30 min. After the centrifugation, the total protein concentrations of the supernatants were quantified using the BCA protein assay kit (Thermo Fisher Scientific, Waltham, MA, USA) according to the manufacturer’s protocol. The extracted proteins (50 µg) were separated and then transferred onto membranes. Antibodies against CYP1A1 (Santa Cruz Biotechnology, Santa Cruz, CA, USA), CYP1B1 (Santa Cruz Biotechnology, Santa Cruz, CA, USA), catalase (Santa Cruz Biotechnology, Santa Cruz, CA, USA), NQO1 (Santa Cruz Biotechnology, Santa Cruz, CA, USA), and β-actin (Santa Cruz Biotechnology, Santa Cruz, CA, USA) were used as the primary antibodies. Anti-rabbit IgG HRP-linked antibody (Cell Signaling Technology, Danvers, MA, USA) and goat anti-mouse IgG-HRP (Santa Cruz Biotechnology, Santa Cruz, CA, USA) antibodies were used as the secondary antibodies. The membranes were visualized using an enhanced chemiluminescence detection reagent (GE Healthcare, Chicago, IL, USA) and Chemi-Doc (Bio-Rad Laboratories, Hercules, CA, USA).

### 2.10. Statistical Analysis

GraphPad Prism 5.0 (GraphPad Software Inc., San Diego, CA, USA) was used for conducting all the statistical analyses. Results are expressed as mean ± SEM from three independent experiments. One-way analysis of variance (ANOVA) and Tukey’s multiple comparison analysis were used to determine the significance of the differences.

## 3. Results

### 3.1. Protective Effect of Curcumin against B[a]P-Induced Cytotoxicity

**Figure 1:**
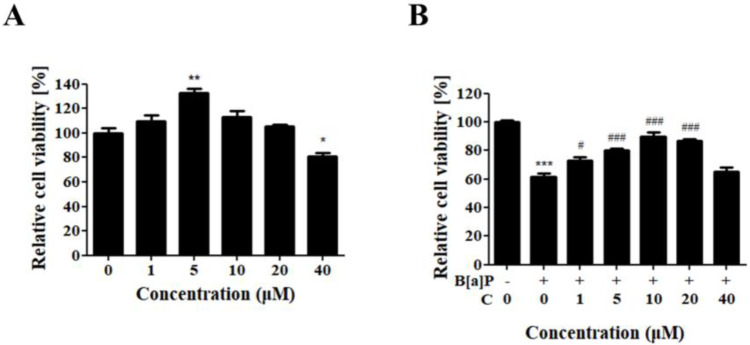
Protective effect of curcumin against B[a]P-induced cytotoxicity. (**A**) Relative viability of HepG2 cells treated with curcumin. *N* = 3 trials per sample and control. (**B**) Ameliorative effect of curcumin on B[a]-induced cytotoxicity in HepG2 cells. *N* = 3 trials per sample and control. * *p* < 0.05, ** *p* < 0.01, and *** *p* < 0.001, compared with the nontreated group; ^#^ *p* < 0.05 and ^###^ *p* < 0.001, compared with the B[a]P-treated group; ^#^ *p* < 0.05 and ^###^ *p* < 0.001, compared with B[a]P-treated cells. B[a]P: benzo[a]pyrene (10 µM); C: curcumin (1, 5, 10, 20, 40 µM).

HepG2 cells were treated with various concentrations of curcumin to evaluate its cytotoxicity on HepG2 cells, and the effect of curcumin against B[a]P-induced cytotoxicity was measured in B[a]P-treated HepG2 cells (Figure 1). HepG2 cells treated with curcumin displayed the highest cell viability at 5 μM and a slightly decreased viability at 40 μM in a statistically significant manner compared with the control. No cytotoxicity was observed up to 20 μM (Figure 1A). Then, the cellular protective effect of curcumin was measured in HepG2 cells cotreated with B[a]P at various concentrations for 48 h. B[a]P treatment decreased the viability of HepG2 cells to 61%, and curcumin treatment recovered their viability progressively up to 89%, showing the highest cell viability at the concentration of 10 μM (Figure 1B). These results suggest that curcumin ameliorates the B[a]P-induced cytotoxicity. Since curcumin showed the highest recovery at the concentration of 10 µM, this concentration was used for further experiments.

### 3.2. Suppressive Effect of Curcumin on the Lipid Accumulation Induced by B[a]P

**Figure 2:**
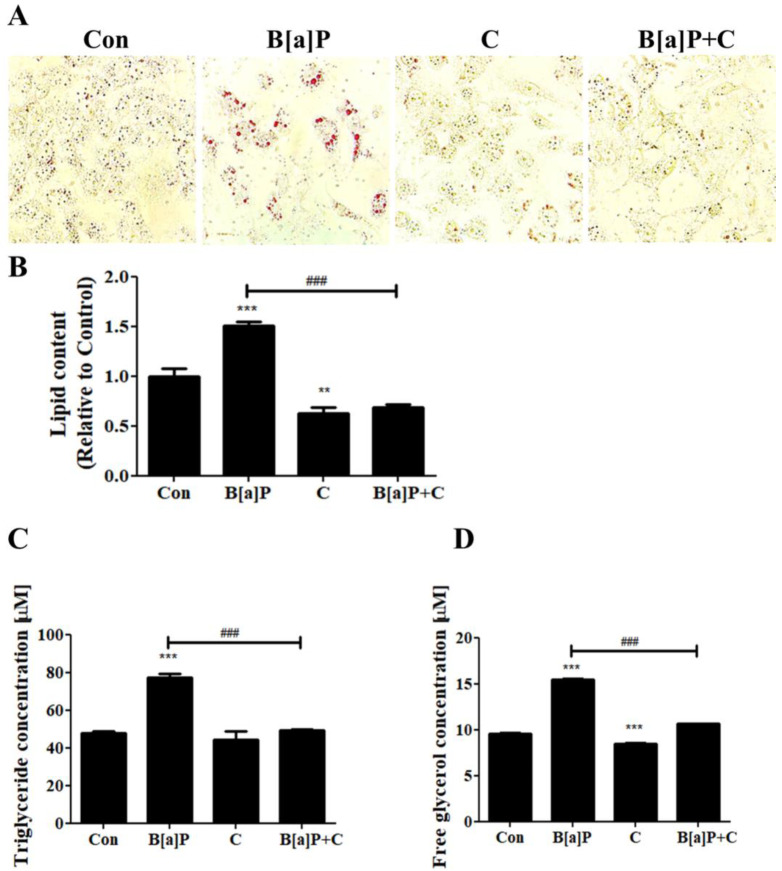
Suppressive effect of curcumin on the lipid accumulation induced by B[a]P. (**A**) Inhibitory effect of curcumin on the B[a]P-induced accumulation of lipid droplets (stained with oil red O). *N* = 5 trials per sample and control. (**B**) Stained lipids were dissolved and quantified. Curcumin-induced attenuation of the increase in the triglyceride (**C**) and free-glycerol (**D**) levels in the cell-culture media. *N* = 3 trials per sample and control. ** *p* < 0.01 and *** *p* < 0.001, compared with the control; ^###^ *p* < 0.001, compared with the B[a]P-treated group. Con: control; B[a]P: benzo[a]pyrene (10 µM); C: curcumin (10 µM).

To evaluate the effect of B[a]P on lipid accumulation and the effect of curcumin on this effect in HepG2 cells, cellular lipids were stained, and cellular triglyceride and free-glycerol levels were measured (Figure 2). We observed that B[a]P treatment induced lipid accumulation in HepG2 cells, and this effect was suppressed by curcumin treatment (Figure 2A). Quantitation of the lipid content of each sample showed increased lipid content in B[a]P-treated cells, which was decreased by curcumin cotreatment (Figure 2B). Curcumin also displayed modulatory effects on triglyceride and free-glycerol levels in the cell-culture media, diminishing the enhancing effects of B[a]P on triglyceride and free-glycerol levels (Figure 2B,C). These results indicate that B[a]P treatment induces lipid accumulation in HepG2 cells and curcumin ameliorates this effect.

### 3.3. Inhibitory Effect of Curcumin on B[a]P-Induced AhR Pathways

**Figure 3:**
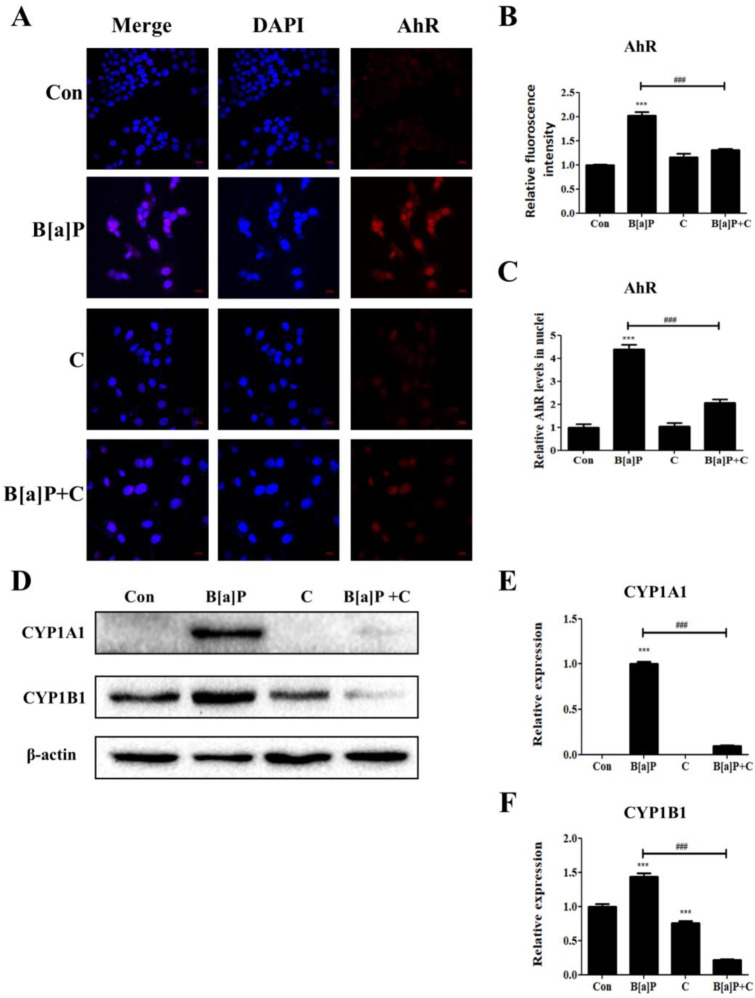
Inhibitory effect of curcumin on B[a]P-induced AhR pathways. (**A**) HepG2 cells were stained for AhR (red). DAPI was used to stain the nuclei (blue). Objective: C-Apochromat 40 ×/ 1.20 W Korr M27. Pinholes: 1 AU for DAPI and AhR. Optical section thickness: 1.2 µm for DAPI and AhR. Modulation of the B[a]P-induced relative fluorescence intensities (**B**) and relative levels of AhR in nuclei (**C**) by curcumin treatment. *N* = 3 trials per sample and control. (**D**) Effect of curcumin on B[a]P-induced upregulation of CYP1A1 and CYP1B1. Relative expression levels of CYP1A1 (**E**) and CYP1B1 (**F**). *N* = 3 trials per sample and control. *** *p* < 0.001, compared with the control; ^###^ *p* < 0.001, compared with the B[a]P-treated group. Con: control; B[a]P: benzo[a]pyrene (10 µM); C: curcumin (10 µM).

B[a]P is well known to be a direct ligand of AhR, which shows transcriptional activity after being translocated into the nucleus [41]. To determine whether the modulatory effects of curcumin on the B[a]P toxicity in HepG2 cells are regulated via the AhR pathways, the fluorescence intensities in the cells upon B[a]P and curcumin treatments were measured via immunostaining methods (Figure 3). The HepG2 cells treated with B[a]P showed an increased fluorescence intensity compared with that of the control, and curcumin cotreatment with B[a]P attenuated the increased expression of AhR in HepG2 cells (Figure 3A,B). The level of nuclear translocation of AhR was also increased upon B[a]P treatment compared with the control, and curcumin cotreatment diminished B[a]P-induced nuclear translocation of AhR (Figure 3A,C). Taken together, these observations indicate that curcumin cotreatment attenuates the B[a]P-induced expression and nuclear translocation of the B[a]P receptor AhR in HepG2 cells.

Upon interaction with its ligand, AhR is translocated into the nucleus and acts as a transcription factor inducing the expression of *Cyp1a1* and *Cyp1b1*, which are involved in the phase-I reactions of drug metabolism and also known as inducers of ROS production [35,38]. The expression of *Cyp1a1* is further induced by cellular ROS, and the increased expression contributes to the cellular lipid accumulation [40]. To investigate whether the curcumin-induced reduction in AhR expression and nuclear translocation in B[a]P-treated HepG2 cells affected the expression of *Cyp1a1* and *Cyp1b1* in these cells, we analyzed the cell extracts for the protein levels of these genes via Western blotting (Figure 3D). CYP1A1 was upregulated by B[a]P treatment but downregulated by the curcumin treatment (Figure 3D,E). The level of CYP1B1 was also increased in B[a]P-treated HepG2 cells, but the expression level was reduced upon curcumin cotreatment (Figure 3D,F). These results indicate that the increased nuclear translocation of AhR by B[a]P induces the expression of *Cyp1a1* and *Cyp1b1*, but this effect is attenuated by curcumin treatment, which contributes to the reduction in ROS generation and inhibition of lipid accumulation in HepG2 cells.

### 3.4. Inhibitory Effect of Curcumin on B[a]P-Induced Nrf2 Pathways

**Figure 4:**
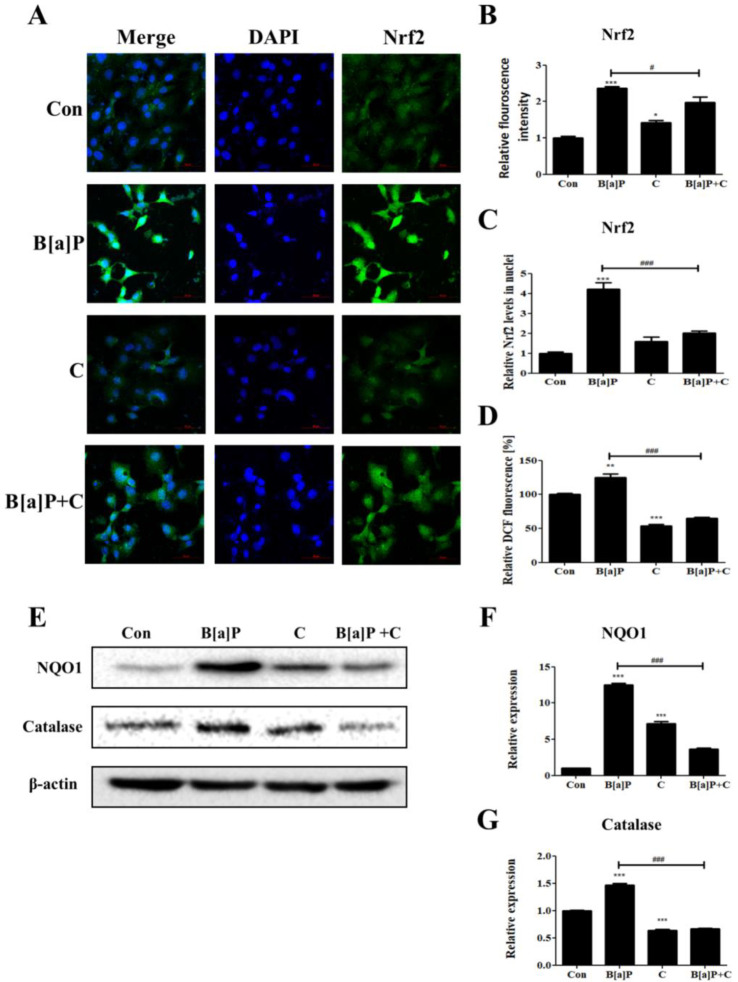
Inhibitory effect of curcumin on B[a]P-induced Nrf2 pathways (**A**) Cells were stained for Nrf2 (green). DAPI was used to stain the nuclei (blue). Objective: C-Apochromat 40×/1.2 W Korr FCS M27. Pinholes: 1.60 AU for DAPI and 1.58 AU for Nrf2. Optical section thickness: 1.5 µm for DAPI and Nrf2. Quantification of the effect of curcumin on the relative fluorescence intensities (**B**) and relative nuclear translocation of Nrf2 (**C**) induced by B[a]P treatment. *N* = 3 trials per sample and control. (**D**) Effect of curcumin on B[a]P-induced upregulation of cellular ROS. *N* = 5 trials per sample and control. (**E**) Effect of curcumin on the levels of phase-II antioxidant enzymes following the increased Nrf2 nuclear translocation. (**F**) Quantification of the levels of the phase-II antioxidant enzymes NQO1 (**F**) and catalase (**G**) in the cells cotreated with curcumin and B[a]P. *N* = 3 trials per sample and control. * *p* < 0.05, ** *p* < 0.01, and *** *p* < 0.001, compared with the control; and ^#^ *p* < 0.05 and ^###^ *p* < 0.001, compared with the B[a]P-treated group. Con: control; B[a]P: benzo[a]pyrene (10 µM); C: curcumin (10 µM).

Nrf2 controls the expression of phase-II antioxidant enzymes and is known to be upregulated and translocated into the nucleus. It is also known that Nrf2 induces the transcription of the *AhR* gene and triggers its downstream pathways [42,43]. To determine whether Nrf2 expression and nuclear translocation are modulated by curcumin in HepG2 cells, the fluorescence intensities in the cells upon B[a]P and curcumin treatments were measured via immunostaining (Figure 4A–C). HepG2 cells treated with B[a]P showed increased fluorescence intensity compared with the control level, but the fluorescence intensity in the B[a]P-treated cells was slightly decreased by curcumin cotreatment (Figure 4A,B). The nuclear translocation of Nrf2 in HepG2 cells increased upon B[a]P treatment, but the curcumin cotreatment attenuated this effect (Figure 4A,C). The B[a]P-induced increase in intracellular ROS level was also attenuated by curcumin (Figure 4D). Phase-II antioxidant enzymes (catalase and NQO1) were upregulated upon the B[a]P treatment in accordance with the elevated nuclear translocation of Nrf2, and the curcumin cotreatment also attenuated this effect (Figure 4E–G). These results suggest that curcumin diminishes the Nrf2 upregulation, nuclear translocation, and subsequent expression of phase-II antioxidant enzymes caused by B[a]P-induced upregulation of ROS.

### 3.5. Effects of Cyp1a1 and Cyp1b1 Knockdown on B[a]P-Induced Cytotoxicity and Induction of ROS

**Figure 5:**
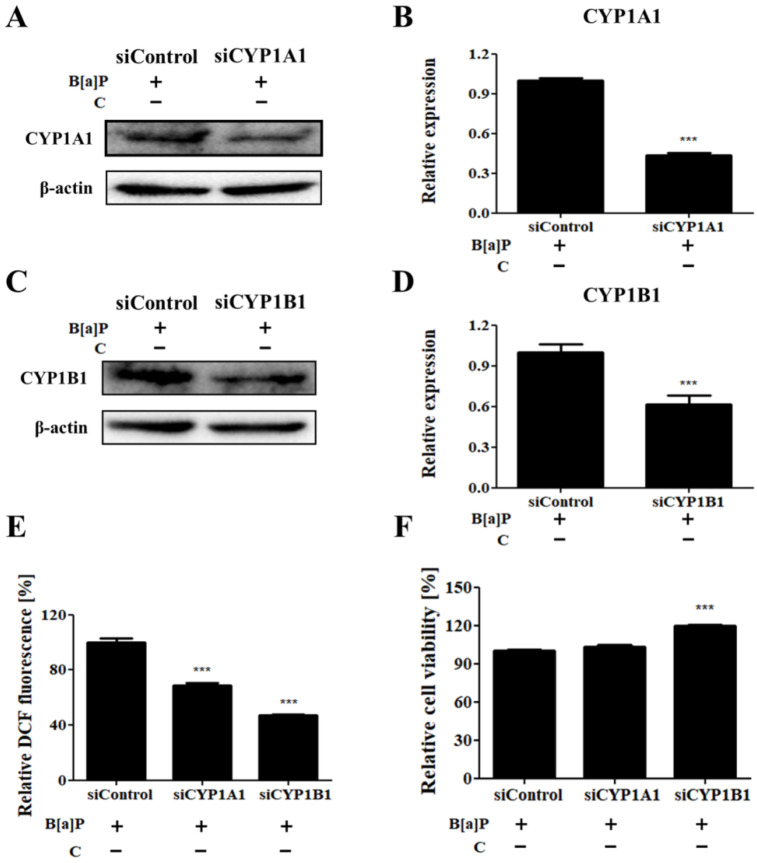
Effects of *Cyp1a1* and *Cyp1b1* knockdown on B[a]P-induced cytotoxicity and induction of ROS. (**A**) *Cyp1a1* knockdown in B[a]P-treated HepG2 cells. (**B**) Relative level of CYP1A1 in siRNA-transfected HepG2 cells compared with the vehicle control. (**C**) *Cyp1b1* knockdown in B[a]P-treated HepG2 cells. (**D**) Relative level of CYP1B1 in siRNA-transfected HepG2 cells compared with the vehicle control. *N* = 3 trials per sample and control. (**E**) Inhibitory effect of *Cyp1a1* and *Cyp1b1* knockdown on cellular ROS generation induced by B[a]P in HepG2 cells. *N* = 5 trials per sample and control. (**F**) Ameliorative effect of *Cyp1b1* knockdown on B[a]P-induced cytotoxicity in HepG2 cells. *N* = 3 trials per sample and control. *** *p* < 0.001, compared with the control. B[a]P: benzo[a]pyrene (10 µM); C: curcumin.

To confirm the modulatory effect of curcumin on CYP450-mediated ROS generation and cytotoxicity induced by B[a]P, CYP1A1 and CYP1B1 levels were downregulated by using specific small interfering RNAs (siRNAs). Knockdown of *Cyp1a1* and *Cyp1b1* was verified using Western blotting (Figure 5A–D). The decreased levels of CYP1A1 and CYP1B1 in HepG2 cells contributed to the attenuation of ROS generation induced by B[a]P (Figure 5E). We also observed that the knockdown of *Cyp1b1* attenuated the cytotoxicity induced by B[a]P treatment in HepG2 cells (Figure 5F). These results suggest that the ameliorative effect on the B[a]P-induced ROS generation in HepG2 cells is mediated by downregulated expression of CYP1A1 and CYP1B1, while cytotoxicity induced by B[a]P is attenuated by CYP1B1 downregulation.

### 3.6. Effect of Cyp1a1 Knockdown on B[a]P-Induced Lipid Accumulation

**Figure 6:**
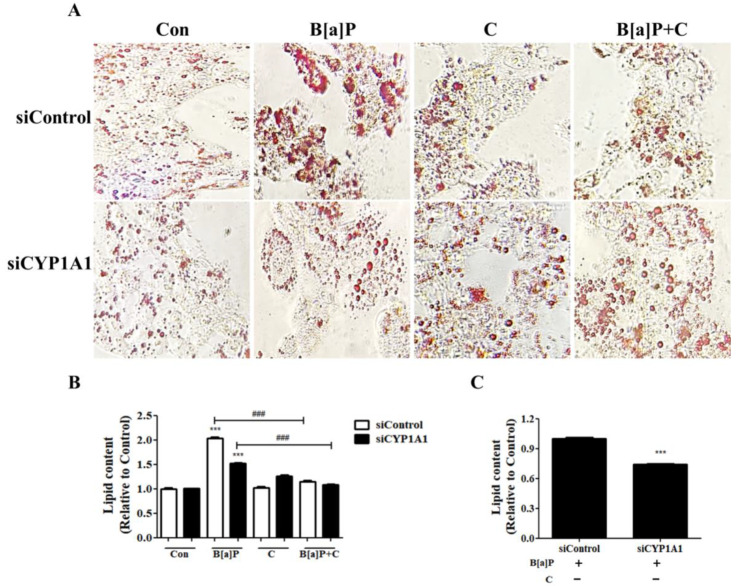
Effect of *Cyp1a1* knockdown on B[a]P-induced lipid accumulation. (**A**) Modulatory effect of decreased expression of CYP1A1 on the accumulation of lipid droplets induced by B[a]P treatment in HepG2 cells. (**B**) Quantitation of the lipid content of HepG2 cells transfected with the vehicle control and *Cyp1a1* siRNA stained with oil red O (ORO). All data were quantified relative to Con in the vehicle control group. (**C**) Suppression of the B[a]P-induced lipid accumulation by *Cyp1a1* knockdown in HepG2 cells. *N* = 3 trials per sample and control. *** *p* < 0.001, compared with the control; ^###^ *p* < 0.001, compared with the B[a]P-treated group. Con: control; B[a]P: benzo[a]pyrene (10 µM); C: curcumin (10 µM).

To examine whether the B[a]P-induced lipid accumulation is dependent on CYP1A1 expression, *Cyp1a1* was downregulated in HepG2 cells by using an siRNA, and the amount of accumulated lipids was measured (Figure 6A). The level of cellular lipids was increased in B[a]P-treated cells compared with the vehicle control group. *Cyp1a1* knockdown attenuated the B[a]P-induced lipid accumulation. The *Cyp1a1*-knockdown cells displayed a significantly lower amount of lipid accumulation upon B[a]P treatment compared with the vehicle control (Figure 6A,C). In addition, lipid content mediated by B[a]P was suppressed by curcumin cotreatment in the vehicle control group, and no additional effect was observed with knockdown of *Cyp1a1* (Figure 6A,B).

## 4. Discussion

B[a]P, listed as a group-1 carcinogen by the IARC, can induce harmful effects on human health. It is found not only in incompletely combusted materials but also in heated and dried foods [16,20]. Consequently, we are unintentionally exposed daily to B[a]P-induced biological damages, and the modulation of B[a]P metabolic pathways to attenuate its cytotoxic effect is important for human health [44].

Curcumin is derived from turmeric (*Curcuma longa*), which is widely used as a health supplement or food flavor [45,46]. Recent studies on the biological activities of curcumin have been mainly focused on the prevention of diseases, including cardiovascular diseases, diabetes, cancer, and obesity [47]. The suppressive role of curcumin on B[a]P-induced toxicity has been well reported but limited to the effects on carcinogenesis and genotoxicity [48,49]. On the other hand, lipid accumulation in hepatocytes possesses the potential to cause hepatic steatosis inducing chronic cell death, while the modulation of the cellular lipid content is required to maintain homeostasis in hepatocytes [29,30,31].

Our study focused on the modulatory effect of curcumin on the B[a]P-induced lipid accumulation and oxidative stress in HepG2 cells, discovering the underlying mechanisms. Curcumin cotreatment recovered the viability of B[a]P-treated HepG2 cells, optimally at the concentration of 10 μM (Figure 1B). Curcumin cotreatment also reduced the intracellular ROS level, which is upregulated by B[a]P, and the recovery of the cell viability upon curcumin cotreatment was correlated with the reduction in intracellular ROS levels (Figure 4D).

Previous studies have shown that increased ROS generation induces lipid accumulation, which leads to further ROS generation, thereby causing obesity and NAFLD [50,51]. In this study, we first demonstrated that B[a]P-treated HepG2 cells exhibited an increased level of lipid droplets, and curcumin diminished such lipid accumulation induced by B[a]P (Figure 2). HepG2 cells cotreated with B[a]P and curcumin displayed fewer lipid droplets than the B[a]P-treated cells, and the levels of intracellular triglyceride and free glycerol in the cell-culture media were also decreased by curcumin treatment (Figure 2A–D). These results indicate that curcumin attenuates lipid accumulation, thereby reducing the B[a]P-induced oxidative stress.

It is well known that B[a]P directly binds to AhR and induces AhR translocation into cell nuclei, mediating the transcription of the *Cyp1a1* and *Cyp1b1* genes [52]. CYP1A1 and CYP1B1 induce ROS generation, and CYP1A1 has recently emerged as a primary regulator of the lipid-droplet production mediated by AhR, which induces lipid accumulation in hepatocytes [35,37,39]. Previous studies have elucidated that curcumin inhibits CYP1A1 and CYP1B1 expression through the regulation of AhR-mediated pathway [53,54]. Our results consistently showed that curcumin cotreatment attenuated the increased AhR expression and nuclear translocation in B[a]P-treated HepG2 cells (Figure 3A–C). The expression levels of CYP1A1 and CYP1B1, which are mediated by AhR nuclear translocation, were increased upon B[a]P treatment, and the cells cotreated with curcumin exhibited reduced expression levels of both CYP1A1 and CYP1B1 (Figure 3D–F).

Nrf2 acts as a major transcriptional factor for phase-II detoxifying enzymes and plays a critical role in drug detoxification in the liver [55]. Nrf2 is functionally repressed, ubiquitylated, and degraded by Keap1 under physiological conditions, and in response to stimuli such as oxidative stress, Nrf2 is liberated from Keap1 and is translocated into the nucleus to activate the transcription of phase-II detoxifying enzymes [56]. Our study showed that Nrf2 expression and nuclear translocation increased upon B[a]P treatment, but these increases were attenuated upon cotreatment with curcumin (Figure 4A–C). A previous study has shown that curcumin inhibits the production of B[a]P metabolites by downregulating CYP1A1 and CYP1B1 [48]. The results indicated that curcumin inhibits the B[a]P metabolism, thereby reducing B[a]P-induced oxidative damages. These observations are consistent with our results showing that the levels of ROS and antioxidant enzymes, such as NQO1 and catalase, in B[a]P-treated HepG2 cells are reduced by curcumin cotreatment (Figure 4D–G).

Recently, crosstalk between AhR and Nrf2 has been revealed. It has been shown that activation of Nrf2 contributes to the upregulation of CYP1A1 and CYP1B1 via inducing the AhR signaling pathway [43]. The results indicated that reduced Nrf2 nuclear translocation inhibits the AhR signaling pathways, thereby downregulating CYP1A1 and CYP1B1; consequently, the B[a]P metabolism is suppressed. Accordingly, the suppressive effect of curcumin on Nrf2 activity in HepG2 cells may have caused the reduction in AhR nuclear translocation. Overall, our observations suggest that curcumin inhibits B[a]P metabolism and then attenuates the cellular lipid accumulation and oxidative stress caused by B[a]P exposure.

Furthermore, knockdown of *Cyp1a1* and *Cyp1b1* in B[a]P-treated HepG2 cells showed that the suppressive effect of curcumin on the B[a]P-induced lipid accumulation and ROS generation is dependent on the expression of CYP1A1 and CYP1B1 (Figure 5 and Figure 6). The B[a]P-induced increase in ROS levels in HepG2 cells is reduced upon knocking down *Cyp1a1* or *Cyp1b1*. The viability of B[a]P-treated HepG2 cells, in which the ROS level had dramatically dropped to 40% compared with the level in the vehicle control, increased upon knocking down *Cyp1b1* (Figure 5E,F). Notably, *Cyp1a1* knockdown approximately to 43% compared to vehicle control attenuated the effect of B[a]P on the lipid accumulation in HepG2 cells, evidenced by the decrease in intracellular lipid contents (Figure 6A,B). However, the accumulated lipid level in the B[a]P-treated group was not recovered to the physiological condition by *Cyp1a1* knockdown. This is because HepG2 cells hardly express CYP1A1 in physiological condition until it is induced by nuclear translocation of AhR [57,58]. Accordingly, the B[a]P-induced lipid accumulation was ameliorated almost down to the physiological condition upon curcumin/B[a]P cotreatment, and no additional effect was observed in the *Cyp1a1*-knockdown cells, which can be explained by the suppressive effect of curcumin on the expression of CYP1A1, as shown in Figure 3D (Figure 6B). Taken together, we demonstrated that the suppressive effect of curcumin on B[a]P-induced lipid accumulation and ROS generation was mediated via the AhR pathway. Consequently, treatment of hepatocytes with curcumin improved maintenance of cell viability against B[a]P-induced cytotoxicity. Our results indicate that curcumin inhibits the expression of CYP1A1 and CYP1B1 through downregulating AhR nuclear translocation, leading to reduced lipid and ROS accumulation. Here, we confirmed that CYP1A1 played a critical role in the lipid accumulation induced by B[a]P and the suppressive effect of curcumin on CYP1A1 expression attenuated the effect, but the underlying mechanisms of the role of CYP1A1 in lipid metabolism are yet to be elucidated.

## 5. Conclusions

In this study, we showed that curcumin cotreatment with B[a]P attenuated cellular lipid accumulation and ROS generation induced by B[a]P through modulating the AhR pathway. Here, in HepG2 cells, we first revealed that the lipid accumulation induced by B[a]P is dependent on CYP1A1, which is attenuated by curcumin treatment. Since B[a]P is naturally ingested via dietary exposure, thereby generating ROS in the human body, the research to uncover detoxifying mechanisms of B[a]P-induced toxicity is important. Our results showing the suppressive effect of curcumin on B[a]P-induced cellular ROS generation and lipid accumulation, which are potential risks toward the development of NAFLD, present a new perspective on the functionality of curcumin and its related therapeutics.

## Data Availability

Data is contained within the article.
